# Tunable rainbow light trapping in ultrathin resonator arrays

**DOI:** 10.1038/s41377-020-00428-y

**Published:** 2020-11-26

**Authors:** Katelyn Dixon, Arthur O. Montazeri, Moein Shayegannia, Edward S. Barnard, Stefano Cabrini, Naomi Matsuura, Hoi-Ying Holman, Nazir P. Kherani

**Affiliations:** 1 Department of Electrical & Computer Engineering, University of Toronto, Toronto, Ontario M5S 3G4 Canada; 2 Lawrence Berkeley National Laboratory, 1 Cyclotron Rd., Berkeley, CA 94720 USA; 3 Department of Materials Science & Engineering, University of Toronto, Toronto, Ontario M5S 3G4 Canada

**Keywords:** Nanophotonics and plasmonics, Sub-wavelength optics, Nanocavities

## Abstract

Rainbow light trapping in plasmonic devices allows for field enhancement of multiple wavelengths within a single device. However, many of these devices lack precise control over spatial and spectral enhancement profiles and cannot provide extremely high localised field strengths. Here we present a versatile, analytical design paradigm for rainbow trapping in nanogroove arrays by utilising both the groove-width and groove-length as tuning parameters. We couple this design technique with fabrication through multilayer thin-film deposition and focused ion beam milling, which enables the realisation of unprecedented feature sizes down to 5 nm and corresponding extreme normalised local field enhancements up to 10^3^. We demonstrate rainbow trapping within the devices through hyperspectral microscopy and show agreement between the experimental results and simulation. The combination of expeditious design and precise fabrication underpins the implementation of these nanogroove arrays for manifold applications in sensing and nanoscale optics.

## Plasmonics: Trapping a rainbow with nanogrooves

A new approach facilitates the fast and versatile design and easy fabrication of nanogroove arrays that can trap the full spectrum of visible light, with potential applications in sensing and nanoscale optics. Katelyn Dixon of the University of Toronto and colleagues conducted mathematical analyses followed by software simulations to determine the wavelengths of visible light that elicit ‘resonance’ within a single metal-insulator-metal nanogroove depending on its width and length. When designed within an array, each nanogroove will have a specific optical response depending on the wavelength of light that causes it to resonate. The team used a unique technique to fabricate devices made of arrays of nanogrooves with different lengths and widths. The narrowest reached five nanometres; ten times smaller than previously designed grooves. The rainbow-trapping arrays overcome limitations in previous designs.

## Introduction

Plasmonic field enhancement in metallic nanostructures is a widely used phenomenon. Applications include sensing via surface enhanced Raman spectroscopy (SERS)^1–6^, plasmon enhanced fluorescence (PEF)^7–9^ and surface enhanced infrared absorption (SEIRA)^10–13^, along with other fields including nonlinear optics^14–17^, super-resolution optics^18–20^ and photo-enhanced catalysis^21–23^. A wide range of nanostructured designs have been developed for these applications using nanoparticles of various shapes and sizes^3,24–26^ and nanocavities in plasmonic materials^8,16,19,27,28^. Typically, these designs consist of arrays of resonators, with resonant wavelengths determined by the size, shape and composition of the individual units^3^. Combining nanostructures with different resonant wavelengths, for example multiresonant nanoparticles^29^ and nanocavities^30–33^, into a single device provides tunable, position-dependent rainbow field enhancement. However, given the lack of analytical solutions for these resonators, it is not feasible to accurately predict the resonant properties of the nanostructures at the design stage without running a large number of iterative detailed simulations. Additionally, the fabrication methods used to date limit the gap sizes within these rainbow trapping structures to 50 nm or larger^33^. As plasmonic field enhancement scales inversely with gap size, this limitation reduces the field strengths which can be realised within these devices^34^. The lack of control over the spectral response of rainbow trapping nanostructures along with the limitation in minimum feature sizes make the efficient, versatile development of devices for various applications a significant challenge.

In this work, we present a framework for fast, versatile, analytical design along with a facile fabrication technique to realise ultrathin rectangular nanogrooves that are subsequently structured into rainbow trapping arrays. The combined advances in both design and fabrication allow for the efficient development of devices tailored for a myriad of applications. We first accurately determined the resonant wavelengths of a single metal-insulator-metal (MIM) nanogroove as a function of the width and length of the groove through analytical calculations. By treating each nanogroove as a Fabry-Perot resonator with a nonnegligible phase shift at the groove boundaries, we can quickly and accurately determine the resonances of a wide range of groove geometries. We then utilise these calculations to design resonator arrays capable of rainbow trapping by varying the width and length of the contained nanogrooves and, for the first time, by varying both parameters together. These arrays are fabricated using a multilayer thin-film deposition and focused ion beam (FIB) milling technique whereby groove-widths as small as 5 nm are attained – an order of magnitude smaller than that reported in previous rainbow trapping studies^31,33^, which provide extremely large localised field enhancements up to 10^3^. Finally, the rainbow trapping capabilities of these devices are demonstrated via far-field hyperspectral microscopy.

## Results

### Analytical design of rainbow trapping groove arrays

**Fig. 1:**
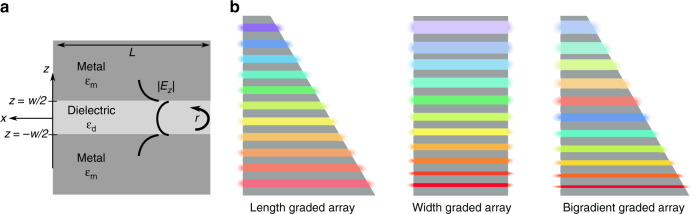
Geometry of MIM groove and illustrations of the three array configurations. **a** Schematic of an MIM groove structure. **b** Illustrations of rainbow trapping in width-graded, length-graded and bigradient groove arrays illustrating the changing resonant wavelength and field intensity with respect to the groove geometry

An ideal MIM waveguide consists of two semi-infinite metallic regions sandwiching a thin dielectric groove which supports the surface plasmon polariton (SPP) mode^35^. Figure 1a illustrates this geometry, where *w* and *L* denote the width and length of the dielectric cavity and *ϵ*_m_ and *ϵ*_d_ represent the dielectric constants in the metal and dielectric, respectively. We focus here on the symmetric mode of the system as it does not experience a cutoff at low values of *w*^35^. The symmetric mode propagates along the length of the groove in the *x* direction until it reaches a free space boundary, at which point it is reflected with reflection coefficient *r*. In a cavity with two open ends, the reflected waves undergo Fabry-Perot resonance subject to the resonance condition

1 $$L = \frac{{m\lambda _{{\mathrm{spp}}}}}{2}$$

where *λ*_spp_ is the plasmon wavelength and *m* = 1,2,3… is the resonant mode order^36^. The resonant condition gives rise to field-intensity maxima within the groove which ultimately contribute to the resulting light trapping. Hence, the ability to accurately predict the resonant modes is essential for the design of effective field-enhancing nanostructures.

A wave reflecting from a dielectric boundary experiences a phase shift of 0 or *π*^37^, however, sufficiently thin dielectric layers (*w* < *λ*) result in a complex reflection coefficient where a phase shift occurs at the boundary^36,38^. This reflection coefficient *r* is given by

2 $$\frac{{1 - r}}{{1 + r}} = \frac{1}{{\lambda \sqrt {\frac{{\mu _o}}{{{\it{\epsilon }}_o}}} \mathop {\int }\nolimits_{\!- \infty }^\infty E_{\mathrm{z}}H_{\mathrm{y}}{\mathrm{d}}z}}\mathop {\int }\nolimits_{ - \infty }^\infty \frac{{\left| {I_1\left( u \right)} \right|^2}}{{\sqrt {1 - u^2} }}{\mathrm{d}}u$$

where *E* and *H* are the electric and magnetic fields inside the groove, $u = \frac{{k_z}}{{k_o}}$, $k_o = \frac{{2{\uppi}}}{{\uplambda }}$, and *I*_1_*(u)* is the Fourier transform with respect to *z*,

3 $$I_1\left( u \right) = {\int} {E_z^{sp}e^{ - ik_ouz}dz}$$

We define the phase of the reflection coefficient according to $r = \left| r \right|e^{i\phi }$^36^. Upon inclusion of this phase shift at the boundary, the modified Fabry-Perot resonance condition becomes^35,36^

4 $$L = \left( {m - \frac{\phi }{\pi }} \right)\frac{{\lambda _{spp}}}{2}$$

The physical cause for this phase shift is the storage of energy in the near field at both ends of the grooves, the magnitude of which increases with the phase shift term, resulting in an increase in the effective length of the groove^36^. This near field energy storage effect makes these nanogrooves ideal for various sensing applications, as light matter interactions can be significantly enhanced when molecules are introduced to the enhanced field at the surface of the groove.

**Fig. 2:**
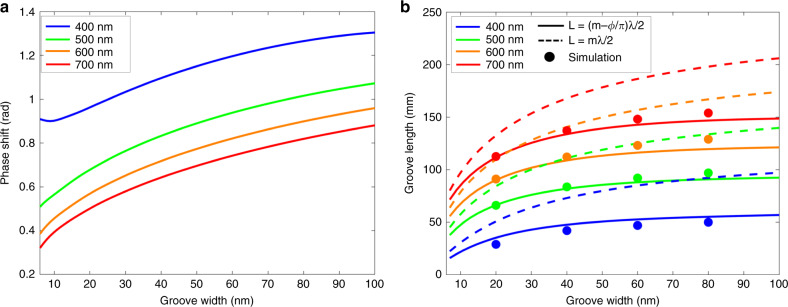
Boundary phase shift and impact on resonant mode in a single groove. **a** Analytically calculated phase shift of an Ag-MgF_2_-Ag cavity with respect to groove width in the visible range. **b** Analytically calculated resonant groove length of the same cavity in relation to the groove width and length with (solid lines) and without (dotted lines) the inclusion of the phase shift φ at the boundary. Numerical simulation data (circular markers) are shown for select geometries. The presented data is for the first resonant mode *m*=1

Figure 2a shows the calculated phase shift as a function of the groove width, while Fig. 2b shows the resonant length of an MIM groove in relation to the cavity width w in the visible range, solved both with and without the phase shift φ. Inclusion of the phase shift reduces the resonant length of the cavity by tens of nanometres, a difference that increases with increasing groove width. To verify the accuracy of these calculations, we used the wave optics module in COMSOL Multiphysics to simulate a two-dimensional rectangular MIM groove at select geometries. The width of the groove and the wavelength were held constant while the groove length was varied until the field intensity within the groove reached a maximum, indicating that the groove was at resonance. Figure 2 shows that the simulation results align with the resonant modes calculated using the phase shift, highlighting the inclusion of the phase shift term as a key component in the accurate design of groove dimensions corresponding to resonance at specific wavelengths. This analytical calculation of the cavity resonant modes, utilising the phase shift term, can be used for expeditious and accurate design of a rainbow trapping device. As shown in Fig. 2, both the length and the width of the groove can be used to tune its resonant wavelength. Specifically, we examine rainbow trapping arrays that result from variations in length and width and by varying both parameters together, as illustrated in Fig. 1b.

### Design and simulation of length-graded arrays

**Fig. 3:**
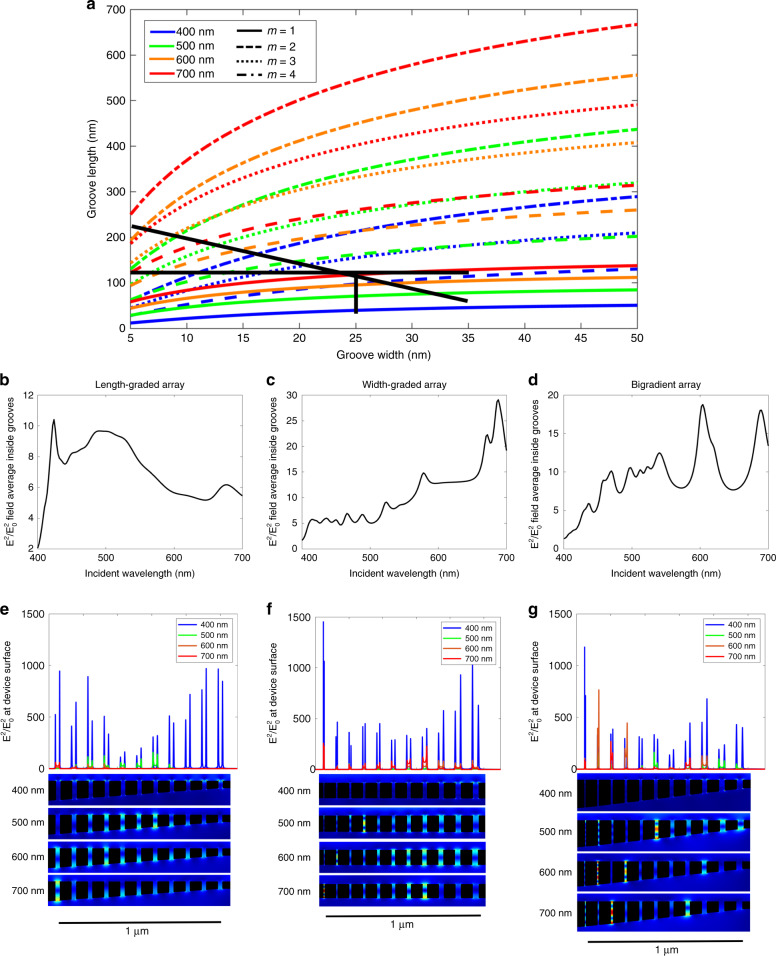
Design of three array configurations and resulting electromagnetic field profiles. **a** Analytical calculation of the first four resonant modes of an Ag-MgF_2_-Ag cavity in the visible regime. Black lines illustrate the groove geometries used in the three array designs. The vertical line represents the length-graded array, the horizontal line represents the width-graded array and the diagonal line represents the bigradient array. **b**–**d** COMSOL simulation of the average field intensity inside the grooves across the entire visible spectrum for the length-graded, width-graded and bigradient designs, respectively. **e**–**g** COMSOL simulation of the electric field intensity at the surface of the array for the length-graded, width-graded and bigradient designs, respectively, at select wavelengths in the visible regime, along with simulated two-dimensional field intensity maps at the four wavelengths

We first examine an array of MIM grooves with a gradient in cavity length. Using Eq. 4, we calculate the first through fourth order resonant mode curves for wavelengths in the visible regime, shown in Fig. 3a. We limit our calculations here to the first four resonant modes as they cover the groove geometries examined in this study, but in studies using larger groove lengths, additional higher order modes can easily be calculated. By tracing a vertical line down this plot, as illustrated in the figure, the range of groove-lengths required to trap the full visible spectrum for a given groove-width is determined. To demonstrate this design technique, we simulate a length-graded groove array composed of 11 grooves of 25 nm width and lengths ranging from 40 to 120 nm with a length gradient of 8 nm per groove, as indicated by the cutline in Fig. 3a. The distance between grooves is 70 nm of Ag while each groove comprises of the dielectric MgF_2_. It should be noted that the groove-to-groove separation can also be used as a tuning parameter to realise rainbow trapping in chirped grating geometries by controlling the SPP dispersion relation^39,40^. As we are tuning the Fabry-Perot resonance with the grooves to obtain rainbow trapping, the groove-to-groove separation is kept constant. The average normalised electric field enhancement within the grooves, shown in Fig. 3b, illustrates that the grooves enhance wavelengths within the visible regime with reasonable uniformity. Figure 3e shows the normalised field enhancement profile for 400, 500, 600 and 700 nm incident wavelength, where the position of maximum field enhancement increases with increasing incident wavelengths, as predicted by Fig. 3a. The one exception to this trend at 400 nm incident wavelength shows two maxima at both long and short groove lengths. The reason is that the maxima at the longer groove length are due to a second order resonant mode, while the short groove maxima is due to a first order mode shown in Fig. 3e. The normalised field enhancements are also very large, with |*E*|^2^/|*E*_o_|^2^ on the order of 10^3^, making this structure ideal for applications requiring strong localised fields^17,19,28^.

Considering the length-graded array presented above, the resonant modes in Fig. 3a dictate the groove length range for rainbow trapping. However, the number of grooves and distance between the grooves (groove-centre to groove-centre) remain free design parameters that can be altered to tune the spatial response of the device as desired. Additionally, these resonant mode calculations can be used to design structures for specific discrete regions of the visible spectrum, thereby making this design technique viable for a large number of applications utilising only a single set of calculations. Further, this technique could also be applied outside the visible spectrum for the design of devices which operate in the near infrared.

### Design and simulation of width-graded arrays

The resonant mode of a groove is dependent on both its width and length as observed in Fig. 2. Groove-width is a very useful design parameter for rainbow trapping structures due to the ease of fabricating width-graded structures as compared to length-graded structures^31,33^. The horizontal line at a groove length of 120 nm shown in Fig. 3a demonstrates that the entire visible range is captured in grooves within the 5–35 nm width range, albeit at various mode orders. To confirm this design, a width-graded array composed of 11 grooves of width 5–35 nm and length 120 nm with a width gradient of 3 nm per groove, is simulated in COMSOL, resulting in the field enhancement profile shown in Fig. 3c. As with the length-graded array, the width-graded array enhances the entire visible spectrum with comparable spectral uniformity and field intensity. Additionally, the number of peaks in the spectrum is larger than that in the length-graded array due to the increased number of trapped modes. Figure 3f shows the field enhancement at the device surface for incident wavelengths of 400, 500, 600 and 700 nm. The peaks in field intensity correlate with the intersection points in Fig. 3a, with first through fourth order modes present among the four wavelengths. The ultrathin groove widths in this design allow for extremely large field enhancements, up to |*E*|^2^/|*E*_o_|^2^ = 1.5 × 10^3^ in the 5 nm groove. As with the length-graded array, this proof of concept design is highly flexible and can be easily optimised for various applications by adjusting the number of grooves or by selecting discrete groove resonances to enhance a specific set of desired wavelengths. For more details on the versatility of width-graded arrays, see the supplementary information.

### Combining width and length gradients

A third possible configuration in a rainbow trapping device is to change both the width and the length of the grooves, forming a ‘bigradient’ device. The diagonal black line in Fig. 3a shows that varying both parameters together allows for a greater number of trapped modes than either width or length-graded arrays. Figure 3d shows the normalised field strength for a bigradient array with the length ranging from 60 to 220 nm and the width ranging from 35 to 5 nm. As predicted by Fig. 3a, the number of peaks is greater than that in both length-graded and width-graded devices, while the field strength remains comparable. Figure 3g shows the field enhancement at the device surface for 400, 500, 600 and 700 nm incident wavelengths. Again, the loci of the maximum field intensity at each wavelength correlate with the intersection points from Fig. 3a. As expected, the field intensity is strongest in the narrowest groove and is comparable to that in the width-graded array. The additional tuning parameters in the bigradient arrays make these bidimensional devices highly adaptable, although the need for precise control over both variables poses significant additional fabrication challenges.

### Fabrication of width-graded and bigradient arrays

**Fig. 4:**
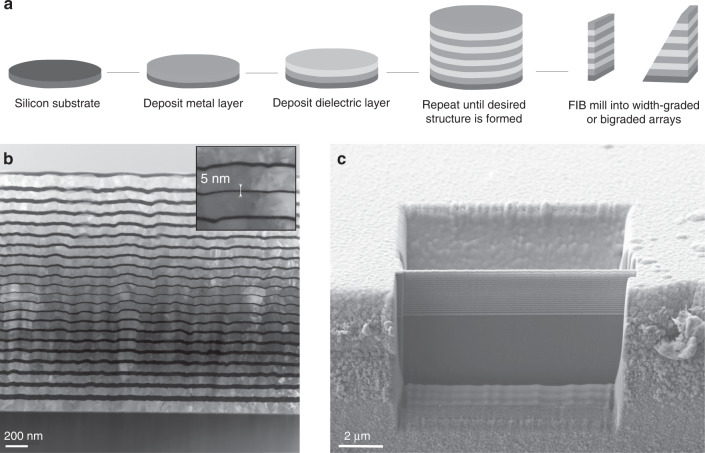
Fabrication of MIM multilayer arrays. **a** Schematic illustration of the fabrication process. **b** TEM image of a symmetric sputter deposited alternating-layer array structure with central groove width of 5nm shown in the inset. The silver and magnesium fluoride layers are light grey and black, respectively. **c** SEM image of a cross sectional slice of the same layered structure milled using FIB. The excavated regions on both sides of the device provide free space boundaries at both ends of the grooves

Ultrathin graded groove arrays are fabricated through radio frequency magnetron sputter-deposition and FIB milling as outlined in Fig. 4a. The width of the MIM grooves is controlled with near single nanometre precision through the deposition of alternating metal and dielectric layers. Subsequently, the FIB is used to define the length of each groove. Considering the extensive set of studies already conducted on length-graded groove arrays^28,30,31,41^, we focus on width-graded and bigradient designs. Silver is used as the metal layer due to its excellent plasmonic properties^42^ and magnesium fluoride is used as the dielectric as its relatively low refractive index optimises mode confinement in the groove. However, both the design paradigm and fabrication techniques can be easily applied to other desired material combinations.

Figure 4b shows a cross sectional transmission electron microscopy (TEM) image of a symmetrically width-graded array with the outermost groove width of 35 nm and central groove width of 5 nm. The ultrathin layers, made feasible by the thin film deposition technique, provide extremely large field enhancements inside the grooves. While the thin films have an inherent surface roughness that increases with the total amount of material deposited, the width of each layer along the vertical axis remains consistent which is the determining factor in the resonant mode of each groove.

**Fig. 5:**
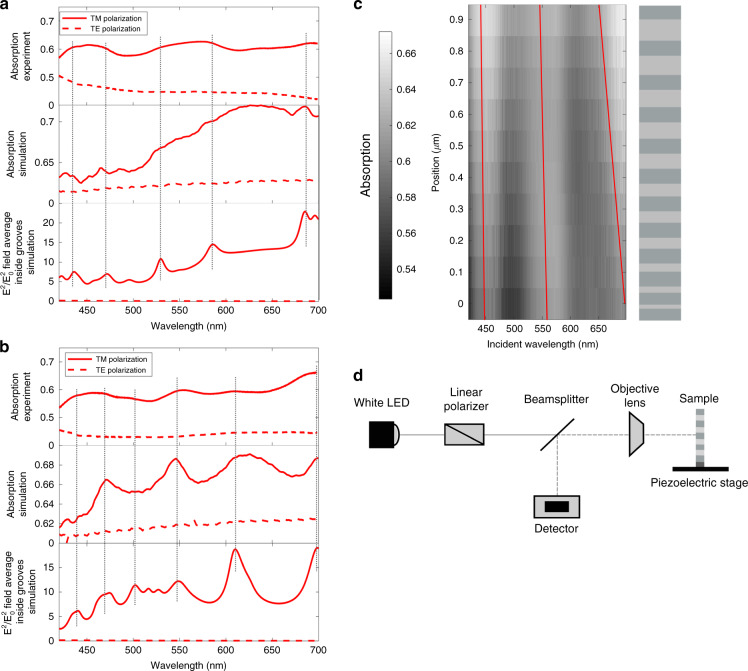
Optical response of graded arrays in the near and far-field. **a**, **b** Experimental and simulated 1−*R* absorption data and simulated |*E*|^2^/|*E*_o_|^2^ electric field data for both TM and TE polarisations for **a** a width-graded array and **b** a bigradient array. **c** Experimental 1−*R* absorption data as a function of array position under TM polarisation for a width-graded array. The schematic illustrates the change in position across the array while the red lines visualise the shifting absorption bands. **d** Schematic illustration of the hyperspectral microscopy apparatus

The FIB is used to create a smooth face at the front of the sample and to mill a region of free space behind the device resulting in a thin MIM stack with the desired Fabry-Perot resonator length. To create a bigradient device, the sample is tilted while milling the back face such that the cut is performed at an angle, creating a gradient in the groove length. Figure 4c shows a bigradient array after FIB milling where the rear cut was performed at a stage tilt of 9°. While a cross sectional image of the devices could not be obtained due to the fragility of the sample, the angle of the cuts was confirmed through comparison to simulations. The spatial and spectral responses of the arrays are measured through far-field hyperspectral microscopy using the apparatus illustrated in Fig. 5d.

### Spectral characterisation of width-graded and bigradient arrays

The average absorption spectra over the device surface are shown in Fig. 5 for both width-graded (Fig. 5a) and bigradient (Fig. 5b) arrays, along with the simulated absorption and near-field electric field intensity inside the grooves. Both the experimental and simulated absorption, approximated as 1−*R* where *R* is the reflection off the sample surface, and near-field strength show a significant decrease in intensity under transverse electric (TE) polarisation. The reason is that SPPs are only excited in the grooves under transverse magnetic (TM) polarisation^35^ resulting in near-field localisation and increased absorption. Under TE polarised illumination, no SPPs are excited and no enhanced modes are observed in either data set, illustrating the contribution of plasmonic resonances to the spectral response.

The absorption spectra of the groove arrays under TM polarisation show a strong correlation with the simulated absorption for both designs, validating the fabrication technique. While the peaks in the experimental data are somewhat broader than those in the simulation data, due to the small size of the sample relative to the field of view, there is generally excellent correlation between the peak locations. Additionally, a correlation between the far-field absorption and near-field enhancement can be observed in both data sets, illustrating the relationship between enhanced absorption and light localisation within the nanostructure. As plasmonically enhanced near fields are typically of significant interest, but near-field measurements can be extremely difficult to perform, the ability to use a far-field measurements from which to infer information about the near field behaviour is very useful.

As predicted by theory and simulation, both width-graded and bigradient groove arrays exhibit rainbow-trapping across the visible spectrum, with the bigradient spectrum containing more peaks than that of the width-graded design. However, there is not a significant improvement in the strength or uniformity of the field enhancement in the bigradient device. Considering the added difficulty of FIB milling a bigradient device, there is a natural appeal for width-graded designs.

### Spatial characterisation of width-graded and bigradient arrays

Figure 5c shows a heat map of the experimental far-field absorption intensity, approximated as 1−*R*, with respect to position on the device surface for a width-graded array. This figure illustrates a blue-shift from long to short wavelengths in the peaks of maximum absorption when moving across the array from narrow to wide groove-widths. This shift is due to changing resonances across the individual grooves, which vary in width from 5 to 35 nm. Figure 3a shows that the resonant wavelength of a groove scales inversely with groove width, such that narrow grooves enhance longer wavelengths than their wider counterparts, resulting in the blue-shift shown in Fig. 5c. While the severity of the shift varies between absorption bands, this is expected as the relationship between the resonance wavelength and groove width is highly nonlinear. If required, this spatial shift could be amplified by increasing the distance between the grooves or by increasing the gradient in the groove-width.

## Discussion

In this study we present a versatile analytical design paradigm for length-graded, width-graded and bigradient rainbow trapping arrays. Using a multilayer thin film deposition process that permits precision groove-width control and focused ion beam milling which defines the groove-length, we demonstrate the fabrication of width-graded and bigradient groove arrays. Through fine control of the facile thin film sputter-deposition technique, a minimum groove width of 5 nm was fabricated, allowing for a significant increase in the field enhancement at the array surface compared to that of existing devices. Optical characterisation through far-field microscopy confirmed rainbow field enhancement across both devices, consistent with near field simulations. The performance of the two example groove arrays is comparable, highlighting the width-graded device as the ideal design due to its relative ease of fabrication. A blue-shift across the 1-µm wide devices is observed in the width-graded array, consistent with the theoretical predictions.

## Materials and methods

Multilayered MIM structures are formed through the sequential sputter-deposition of metal and dielectric thin films. Silicon, with the native oxide layer removed through hydrofluoric acid etching, is used as a substrate due to its unparalleled smoothness. During deposition, the substrate is rotated at 20 rpm to ensure even film thickness. Initially, a 10-nm layer of chromium is deposited to improve the adhesion between silver and the underlying silicon. Next, the first silver layer is deposited followed by the deposition of the first dielectric layer. This process is repeated until the desired number of layers is formed, which in the present case is 12 silver and 11 dielectric layers in total. The sputtering parameters on the Kurt J Lesker multitarget sputtering facility are optimised to minimise the roughness of the layers, while the layer thickness is controlled with near single nanometre precision.

A Ga^+^ FIB ZEISS Crossbeam is used for milling the Ag-MgF_2_-Ag multilayer stack. FIB milling is a high-resolution tool for shaping matter at the nanometre scale and has the ability to sculpt matter in more than one dimension. While heterogeneity of materials such as the stack of alternating metal and dielectric layers poses a challenge to other techniques, the FIB can cut through the stack with ease and precision. Paired with SEM capabilities, the quality of devices can be inspected in situ providing real-time feedback on the quality of nano-milled structures. In addition, automatic stage control for milling large numbers of structures even on a wafer-scale, provides a high-precision, reproducible, and high-yield rapid prototyping approach. Before milling, 50 nm of titanium was evaporated onto the top of the structure to reduce ion implantation during milling. A milling current of 50 mA was used to remove large amounts of material behind the device, while a 10-mA milling current was used to define the edges of the device, minimising ion implantation.

The field enhancement within the groove arrays is characterised through reflection mode hyperspectral microscopy. The optical arrangement, pictured in Fig. 5d, passes linearly polarised light through a beamsplitter and ×100 objective lens onto the sample mounted on a piezoelectric stage. The reflectivity is measured over the entire sample surface with measurements taken every 100 nm in both spatial dimensions. Measurements are carried out under both TE and TM polarisations to characterise the effects of plasmonic field enhancement within the device.

## Supplementary information

Supplemental Material

## Data Availability

All code, simulation, and data files used to obtain the described results are available from the authors upon request.
