# Supplementary material for: Tunable rainbow light trapping in ultrathin resonator arrays
Source: Light Sci Appl. 2020 Nov 26;9:194. doi: 10.1038/s41377-020-00428-y (PMC7693327; doi:10.1038/s41377-020-00428-y)
Supplement: Supplementary file 1 — Supplemental Material [file 41377_2020_428_MOESM1_ESM.docx]

Supplementary information for tunable rainbow light trapping in ultrathin resonator arrays

Katelyn Dixon^1^, Arthur O. Montazeri^2^, Moein Shayegannia^3^, Edward S. Barnard^4^, Stefano Cabrini^5^, Naomi Matsuura^6^, Hoi-Ying Holman^7^, and Nazir P. Kherani^8*^

^1^Department of Electrical & Computer Engineering, University of Toronto, Toronto, Ontario, M5S 3G4, Canada. E-mail: katelyn.dixon@mail.utoronto.ca

^2^Lawrence Berkeley National Laboratory, 1 Cyclotron Rd., Berkeley, CA, 94720, USA. E-mail: arthur.montazeri@gmail.com

^3^Department of Electrical & Computer Engineering, University of Toronto, Toronto, Ontario, M5S 3G4, Canada. E-mail: moein.shayegannia@mail.utoronto.ca

^4^Lawrence Berkeley National Laboratory, 1 Cyclotron Rd., Berkeley, CA, 94720, USA. E-mail: esbarnard@lbl.gov

^5^Lawrence Berkeley National Laboratory, 1 Cyclotron Rd., Berkeley, CA, 94720, USA. E-mail: scabrini@lbl.gov

^6^Department of Materials Science & Engineering, University of Toronto, Toronto, Ontario, M5S 3G4, Canada. E-mail: naomi.matsuura@utoronto.ca

^7^Lawrence Berkeley National Laboratory, 1 Cyclotron Rd., Berkeley, CA, 94720, USA. E-mail: hyholman@lbl.gov

^8^Department of Materials Science & Engineering and Department of Electrical & Computer Engineering, University of Toronto, Toronto, Ontario, M5S 3G4, Canada. E-mail: nazir.kherani@utoronto.ca Telephone: 416-618-2654

**Design of arrays for single and multi-wavelength field enhancement**

In order to demonstrate the precision design capabilities of the analytical paradigm, we have designed and simulated three groove arrays for the enhancement of a single wavelength, two wavelengths, and three wavelengths. Figure S1a shows the first order resonant modes for four wavelengths in the visible regime. We target the first order modes here as designing grooves with these geometries reduces the presence of higher order modes which broaden the spectral response. We first design an array for the enhancement of a single wavelength, 500 nm, selecting a groove length of 60 nm and groove width of 16 nm as illustrated in Figure S1a. We then simulate an array composed of twelve identical 60 by 16 nm magnesium fluoride grooves each separated by 100 nm of silver. Figure S1b and e show the spectral and spatial field enhancement within this array, with an enhancement peak at 500 nm. We can similarly design an array to enhance two select wavelengths, in this case 500 and 600 nm, by adding grooves with a length of 60 nm and a width of 8 nm, as shown in Figure S1a. We then simulate an array of 12 grooves with length 60 nm and width alternating between 16 and 8 nm of magnesium fluoride, with 100 nm of silver in between. The resulting field enhancement is shown in Figure S1c and f, with enhancement peaks at 500 and 600 nm. Lastly, we expand this technique to enhance a third wavelength, 700 nm, by incorporating grooves of length 60 nm and width 5 nm into the array. The field enhancement, shown in Figure S1d and g, shows peaks at all three wavelengths. This design technique can be adapted to enhance any number of select wavelengths within a width-graded array. Additionally, the spatial response can be altered simply by changing the order of the grooves of different widths, and the spectral response can be adjusted by changing the number of grooves of each dimension. While we have used a width-graded array to demonstrate this principle, similar techniques can be used to design multi-wavelength arrays with a length gradient or bigradient.


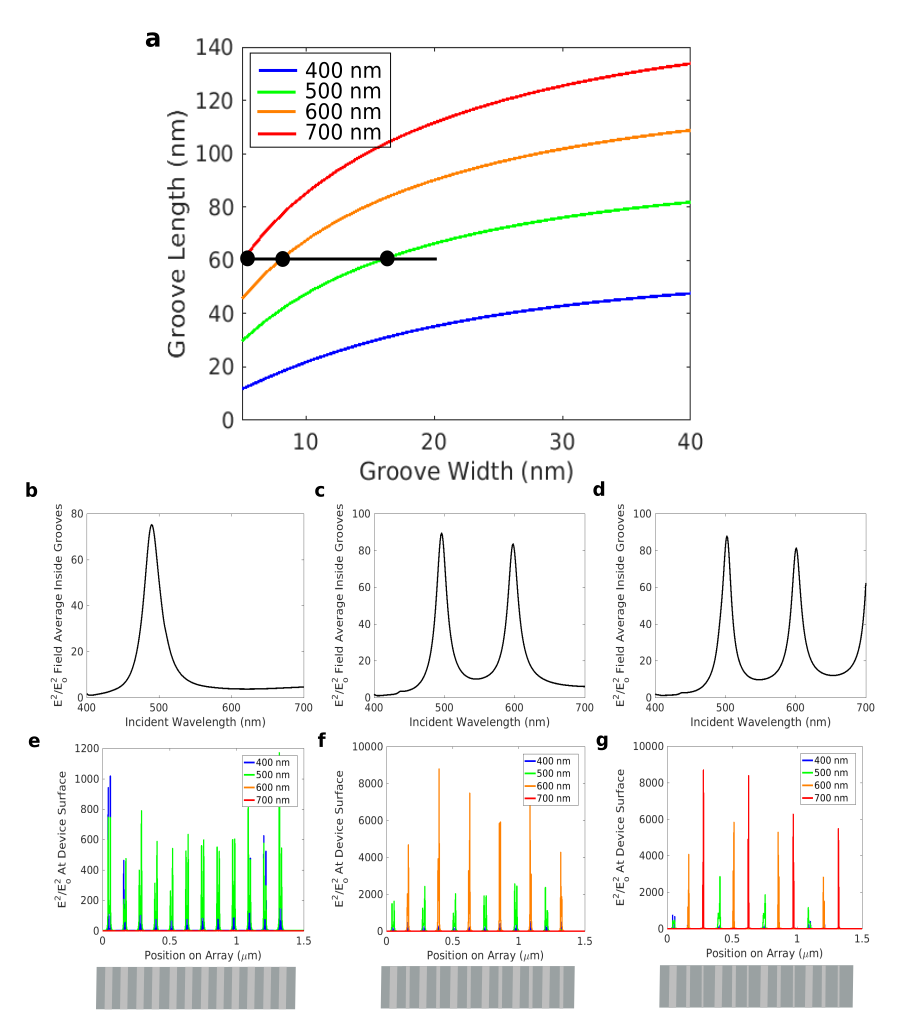


Figure S1: (a) Analytical calculation of the first resonant mode of an Ag-MgF_2_-Ag cavity in the visible regime. Black circles represent the three groove geometries used in the simulations. (b, c, d) COMSOL simulation of average field intensity inside the grooves across the entire visible spectrum for (b) single wavelength, (c) two wavelength, and (d) three wavelength enhancing arrays. (e, f, g) COMSOL simulation of electric field intensity at the surface of the three arrays at select wavelengths in the visible regime along with illustrations of the array geometries.

**Effect of the number of grooves in an array**

While we have so far dealt with arrays containing a constant number of grooves, increasing the number of grooves in a given array broadens the spectral response by increasing the number of resonators and decreasing the resonance gradient. To demonstrate this, we simulated three rainbow trapping arrays with a groove length of 120 nm and groove widths ranging from 5 to 35 nm of magnesium fluoride, with 70 nm of silver in between, as illustrated in Figure S2a. The number of grooves was set to 10, 20 and 30, with the gradient in the groove width adjusted to span the 5 to 35 nm range in each case. Figure S2b shows the spectral response of each array in which it can be seen that the number of peaks increases with the number of grooves, gradually broadening the field enhancement. While ultra-broad uniform field enhancement is advantageous for many applications, it requires a larger total array size, which often increases the time and cost involved in fabrication. Additionally, increasing the number of grooves reduces the spatial uniformity, which may not be compatible with applications which require illumination with a small focal area which may not encompass the entire array and therefore will not excite the full spectral response. All these considerations must be taken into account when designing rainbow trapping arrays for various applications.


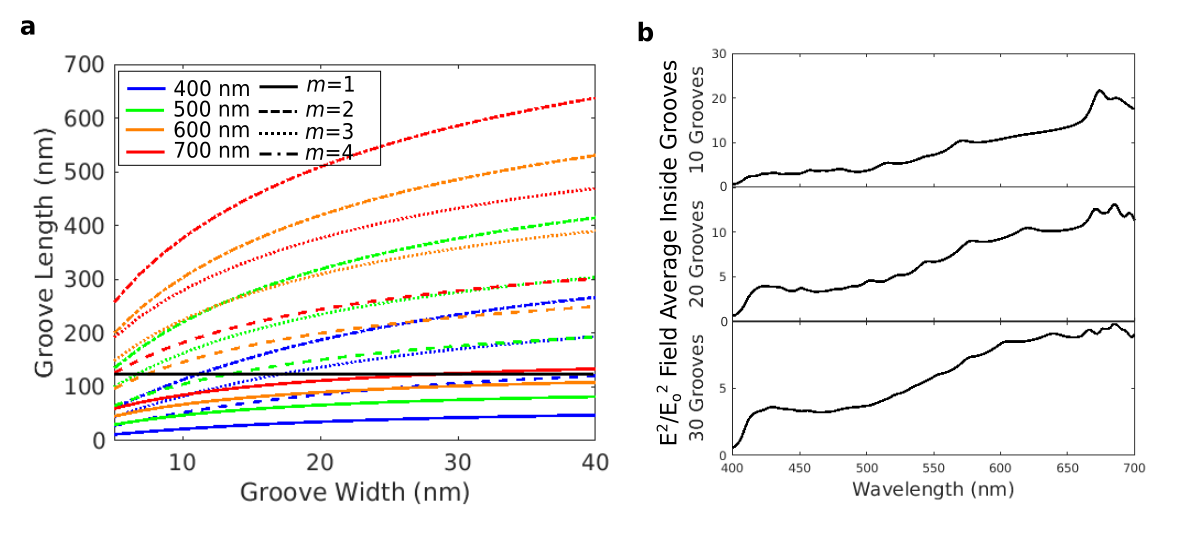


Figure S2: (a) Analytical calculation of the first four resonant modes of an Ag-MgF_2_-Ag cavity in the visible regime. The black line illustrates the groove geometries used in the three array designs. (b) COMSOL simulation of the average field intensity inside the grooves across the visible spectrum for arrays composed of 10 grooves, 20 grooves and 30 grooves.
